# DNA methylation and differential gene regulation in photoreceptor cell death

**DOI:** 10.1038/cddis.2014.512

**Published:** 2014-12-04

**Authors:** P Farinelli, A Perera, B Arango-Gonzalez, D Trifunovic, M Wagner, T Carell, M Biel, E Zrenner, S Michalakis, F Paquet-Durand, P A R Ekström

**Affiliations:** 1 Division of Ophthalmology, Department of Clinical Sciences, University of Lund, BMC-B11, Lund 22184, Sweden; 2 Division of Experimental Ophthalmology, Institute for Ophthalmic Research, University of Tübingen, Tübingen 72076, Germany; 3 Center for Integrated Protein Science Munich (CIPSM) at the Department of Pharmacy – Center for Drug Research, Ludwig-Maximilians-Universität München, Munich 81377, Germany; 4 Center for Integrated Protein Science Munich (CIPSM) at the Department of Chemistry, Ludwig-Maximilians-Universität München, Munich 81377, Germany

## Abstract

Retinitis pigmentosa (RP) defines a group of inherited degenerative retinal diseases causing progressive loss of photoreceptors. To this day, RP is still untreatable and rational treatment development will require a thorough understanding of the underlying cell death mechanisms. Methylation of the DNA base cytosine by DNA methyltransferases (DNMTs) is an important epigenetic factor regulating gene expression, cell differentiation, cell death, and survival. Previous studies suggested an involvement of epigenetic mechanisms in RP, and in this study, increased cytosine methylation was detected in dying photoreceptors in the *rd1*, *rd2*, P23H, and S334ter rodent models for RP. Ultrastructural analysis of photoreceptor nuclear morphology in the *rd1* mouse model for RP revealed a severely altered chromatin structure during retinal degeneration that coincided with an increased expression of the DNMT isozyme DNMT3a. To identify disease-specific differentially methylated DNA regions (DMRs) on a genomic level, we immunoprecipitated methylated DNA fragments and subsequently analyzed them with a targeted microarray. Genome-wide comparison of DMRs between *rd1* and wild-type retina revealed hypermethylation of genes involved in cell death and survival as well as cell morphology and nervous system development. When correlating DMRs with gene expression data, we found that hypermethylation occurred alongside transcriptional repression. Consistently, motif analysis showed that binding sites of several important transcription factors for retinal physiology were hypermethylated in the mutant model, which also correlated with transcriptional silencing of their respective target genes. Finally, inhibition of DNMTs in *rd1* organotypic retinal explants using decitabine resulted in a substantial reduction of photoreceptor cell death, suggesting inhibition of DNA methylation as a potential novel treatment in RP.

Retinitis pigmentosa (RP) refers to a heterogeneous group of inherited retinal degenerations that provoke progressive and irreversible loss of photoreceptors. In the developed countries, RP represents the leading cause for severe vision loss and blindness among young people.^1^ Currently, mutations in >50 genes have been linked to RP (https://sph.uth.tmc.edu/retnet/), but the mechanisms that lead to photoreceptor death are still unresolved, and there is no treatment available. RP therapy development is severely limited by the high genetic heterogeneity, which therefore calls for the identification of common disease mechanisms and drug targets.

A previous microarray study showed a dramatic modification in gene expression when the *rd1* mouse model for RP was compared with wild-type (*wt*) animals.^2^ Such profound effects on gene expression are probably a consequence of both pro-survival responses and induction of cell death pathways. Alterations of gene expression are often linked to epigenetic events, such as acetylation and poly-ADP-ribosylation of histones. In this regard, we have previously found the activity of both histone deacetylase (HDAC) and poly-ADP-ribose-polymerase (PARP) to be causally involved in retinal degeneration.^3, 4^ Although these epigenetic regulators function via modification of the chromatin structure, direct methylation of the DNA^5, 6^ is another powerful factor in epigenetic regulation of gene expression. DNA methylation was recently connected with retinal degeneration and retinal development.^7^ However, it is still unknown if and how DNA methylation patterns vary between different genes in healthy and diseased tissues and whether an interference with DNA methylation would be beneficial for degenerating photoreceptors.

The covalent addition of a methyl group on cytidines followed by guanosine in the DNA is performed by various DNA methyltransferases (DNMTs) such as DNMT1, DNMT3A, and DNMT3B,^8, 9^ while DNMT3L may stimulate *de novo* DNA methylation.^10^ Methylation occurs mainly in gene promoters but can also be seen in intergenic non-coding regions and within genes.^11^ DNA methylation is generally associated with repression of transcription.^12^

Here, we have compared retinal DNA methylation in the *rd1* mouse model,^13^ with those of healthy, congenic *wt* mice and correlated DNA methylation changes with gene expression data sets. We then used an organotypic retinal explant system to evaluate the effect of a DNA methylation inhibitor on *rd1* photoreceptor survival *in vitro*. We furthermore analyzed the extent of DNA methylation in three other RP models, namely the *rd2* mouse and the S334ter and P23H transgenic rats. All four RP models represent mutations that are similar to the ones found in certain cohorts of patients.^14, 15, 16^

Our data show an increase in DNA methylation in dying photoreceptors in all four RP animal models analyzed, suggesting DNA hypermethylation as a common denominator in the photoreceptor degeneration pathway. Moreover, our results reveal that in *rd1* retinae specific genes may be either hypomethylated or hypermethylated. Pharmacological inhibition of DNMTs significantly reduced *rd1* photoreceptor cell death in short-term experiments but did not increase cell survival in the long-term experiments. Our findings thus suggest a complex relation between DNA methylation and retinal degeneration, which may include both disease-driving and disease-counteracting elements.

## Results

### *rd1* photoreceptors show abnormal chromatin structure

**Figure 1:**
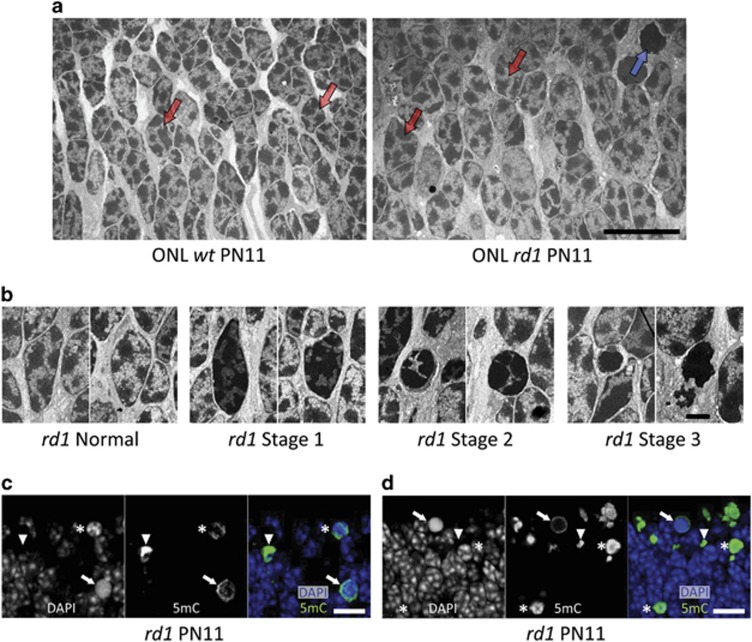
Altered *rd1* nuclear ultrastructure and DNA methylation. (**a**) The overview of PN11 *wt* and *rd1* ONL illustrates the mixed distribution of heterochromatin and euchromatin (dark and light areas, respectively) in both *wt* and (most) *rd1* photoreceptor nuclei. Typical examples of such nuclei are pointed out by red arrows. By contrast, *rd1* photoreceptor nuclear configurations varied considerably from ‘normal' (=similar to *wt* nuclei), via the different stages 1 and 2, to very condensed, electron dense and dark, that is, stage 3 (blue arrow in *rd1* picture). These stages are shown in more detail in panel (**b**). (**c** and **d**) In *rd1* PN11 retina, immunostaining for 5mC (green), together with a nuclear counterstain (4,6-diamidino-2-phenylindole (DAPI), blue), showed varying degrees of co-localization. 5mC-positive structures had a DAPI appearance that was either heterogeneous rounded (*), or homogenous rounded (arrows), or weak to the point of being absent (arrowheads), most likely reflecting the different stages of nuclear condensation identified in panel (**a**). The confocal images in **c** and **d** are maximum projections of 16 and 21 *Z*-sections, respectively. Scale bars: **a**, **c**, and **d**=10 *μ*m, **b**=2 *μ*m

Epigenetic modifications, such as DNA methylation, manifest themselves in chromatin rearrangements. For an initial assessment, we performed an analysis of photoreceptor nuclei ultrastructure in PN11 *rd1* and *wt* retinae. Nuclei of both *rd1* and *wt* photoreceptors (Figure 1a) contain both dense heterochromatin (dark in the EM micrographs) with low transcriptional activity and loose euchromatin (light in the micrographs) with high transcriptional activity. This is normal for mouse photoreceptor nuclei at this age, although adult rod nuclei in contrast to other cells display an inversed nuclear architecture, that is, their euchromatin is collected closest to the nuclear envelope and the heterochromatin placed in the center of the nuclei.^17^

A subset of *rd1* photoreceptor nuclei had a different appearance, and their chromatin and nuclear characteristics could be roughly categorized into four general stages (Figure 1b). Initially, *rd1* photoreceptor nuclei appear normal, comparable to *wt*, with a clear density difference between heterochromatin and euchromatin, and with ‘fuzzy' borders of the heterochromatin. *rd1* nuclei, which had an elongated form and normal size but displayed a darker euchromatin and sharper heterochromatin borders, were categorized as Stage 1. *rd1* nuclei, with a smaller and rounded appearance, and with proportionally much more heterochromatin, with sharp borders were labeled as Stage 2. Some stage 2 nuclei also showed a euchromatin-containing area at their rim (Figure 1b). Stage 3 nuclei were in principle homogenously dark with only heterochromatin and were either round or irregular in shape and much smaller than normal nuclei. Stage 3 may represent the final phase of photoreceptor death, as they resemble the small terminal-dUTP-nick-end-labeling (TUNEL)-positive cells frequently observed in *rd1* specimens of this age (see, e.g., Figure 4 in Sancho-Pelluz *et al.*^4^). Taken together, the nuclear structure of the degenerating *rd1* photoreceptors is severely altered.

### DNA methylation is increased in dying photoreceptors

To study photoreceptor DNA methylation *in situ*, we used immunofluorescence with an anti-5-methylcytosine (5mC) antibody. 5mC-positive *rd1* nuclei were most often round and also frequently smaller than their negative counterparts (Figures 1c and d). Combined with the EM data, this could indicate that DNA hypermethylation occurs primarily at later stages of *rd1* photoreceptor death (i.e., Stages 2 and 3 in Figure 1b). The 5mC signal was commonly localized to the nuclear rim (arrows in Figures 1c and d) or had a very condensed appearance (arrowheads in Figures 1c and d). Remarkably, 5mC appeared to avoid the most DAPI-dense parts of the nuclei, including supposed chromocenters (asterisk in Figure 1c). As DAPI is a dye that binds to the minor groove of the DNA,^18^ 5mC-positive DNA may have lost its conventional confirmation and minor groove resulting in a DAPI-negative staining.

**Figure 2:**
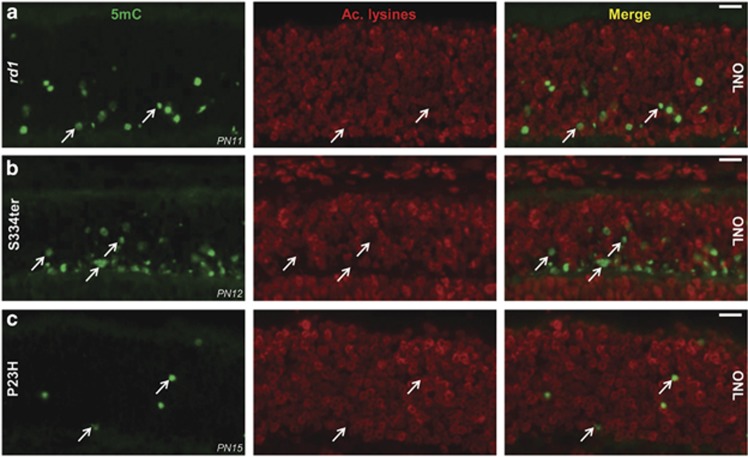
DNA methylation colocalizes with protein deacetylation. Loss of lysine acetylation can mark HDAC activation (Sancho-Pelluz *et al.*^4^). Stainings for 5mC and lysine acetylation (Ac. lysines) were here combined, which resulted in that 5mC staining often occurred in nuclei where acetylated lysines were very low or absent, suggesting an interplay between HDAC and DNMT activities. This was seen in (**a**) *rd1* mouse, as well as in (**b**) S334ter and (**c**) P23H rat retinae. ONL: the individual figures are oriented such that this layer is up and inner retinal layers are down. Scale bar=20 *μ*m. The results are representative for observations made in at least three different individuals of each genotype

In *rd1* retina, alterations of nuclear morphology correlate with increased HDAC activity,^4^ and we therefore assessed protein acetylation together with DNA hypermethylation. Interestingly, three different rodent RP models (*rd1* mouse, S334ter, and P23H rats) displayed 5mC labeling in cells with lysine hypoacetylation (Figure 2), that is, with overactivated HDACs,^4, 16^ indicating that more than one epigenetic event may simultaneously affect degenerating photoreceptors.

**Figure 3:**
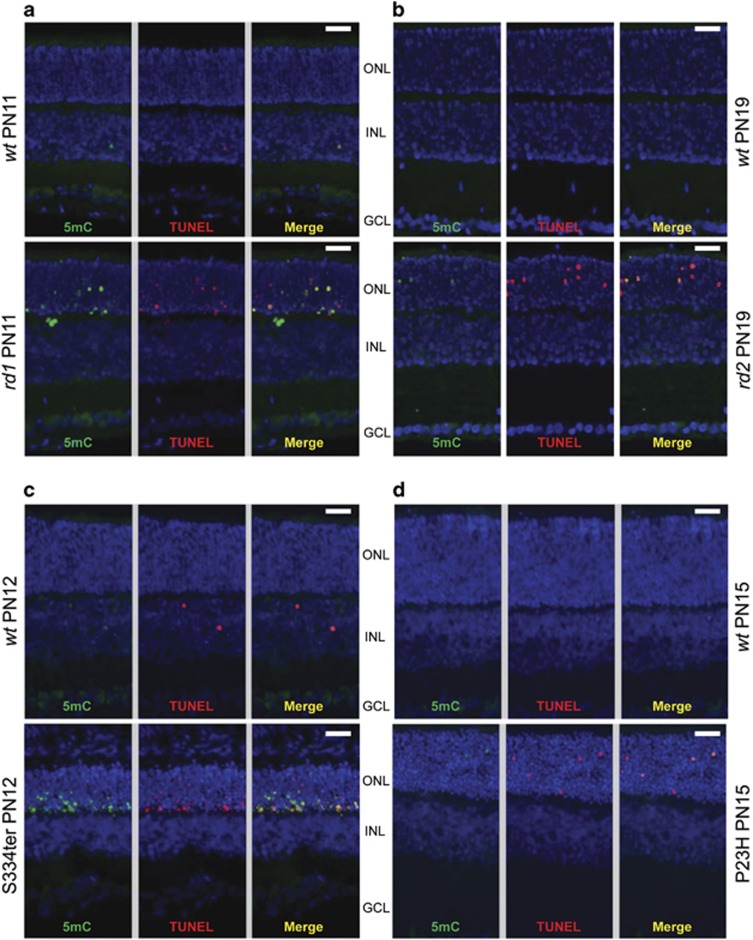
DNA methylation colocalizes with cell death in four different RP animal models. An immunostaining for 5mC was performed together with the TUNEL assay for dying cells in four animal models for RP. In these models, co-staining was performed at time points corresponding to the onset or peak of retinal degeneration. In (**a**) PN11 *rd1* and (**b**) PN19 *rd2* mouse retina, as well as in (**c**) PN12 S334ter and (**d**) PN19 P23H rat retina, the numbers of 5mC immunodecorated photoreceptors was increased when compared with *wt* controls and most of the 5mC-positive cells were co-labeled with the TUNEL assay. GCL=ganglion cell layer; INL=inner nuclear layer. Scale bar=20 *μ*m. The results are representative for observations made in at least three different individuals of each genotype

Strong immunoreactivity for methylated DNA was only rarely found in any retinal cell layer of *wt* animals or in the inner retinal layers in *rd1* animals. By contrast, a distinct subset of cells in the *rd1* outer nuclear layer (ONL) had clearly labeled nuclei (Figure 3a), that were observed also in other models for RP, including the *rd2* mouse. In all of these models, and in line with the *rd1* data, a sub-population of photoreceptors showed an increase of nuclear DNA methylation at the evaluated time points compared with *wt* counterparts (Figures 3b–d). This suggested increased DNA methylation as a general phenomenon during inherited photoreceptor degeneration.

In all the analyzed models, the TUNEL assay for dying cells co-labeled the 5mC-positive cells to a great extent, suggesting an intimate connection between increased DNA methylation and the degeneration of the photoreceptors (Figures 3a–d). Moreover, the various models have different degeneration kinetics, which leads to different numbers of dying photoreceptor cells at their respective peak of degeneration.^16, 19, 20^ Yet, the amount of 5mC-positive ONL cells reflected the amount of TUNEL-positive cells faithfully (Figures 3a–d), again indicating that the DNA hypermethylation was somehow related to the degeneration.

**Figure 4:**
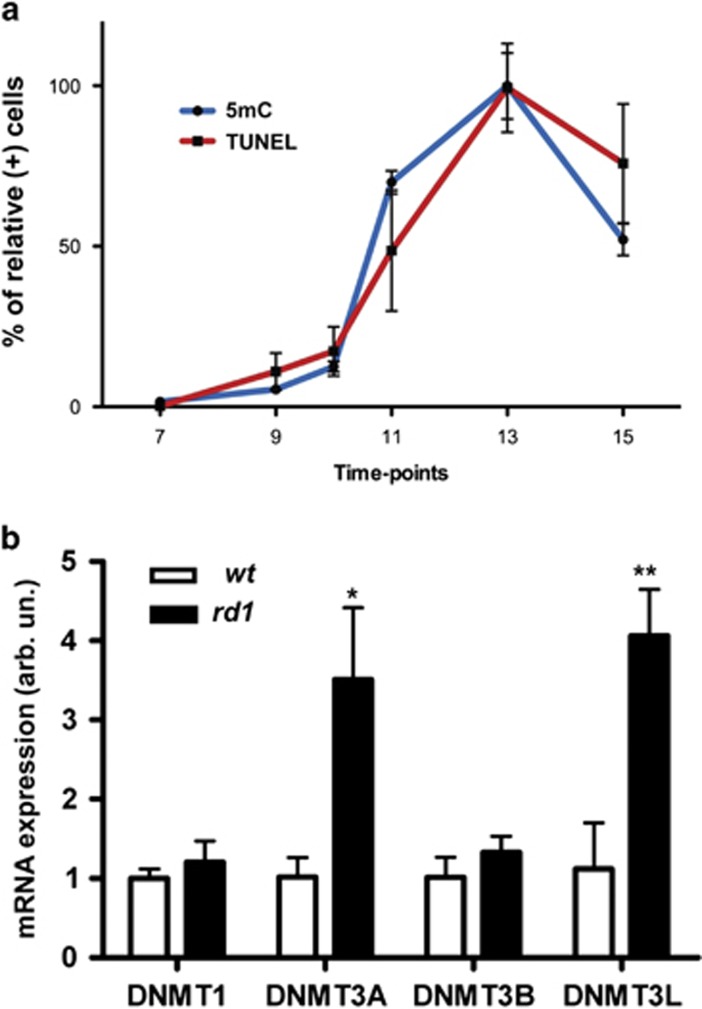
Temporal dynamics of DNA methylation and DNMT gene expression. (**a**) In *rd1* ONL, at early PN ages, the numbers of photoreceptors showing 5mC staining were very low, increased from PN11 onwards, and peaked at PN13. The temporal progression of 5mC positivity in *rd1* ONL corresponded largely to the extent of cell death as evidenced by TUNEL staining, implying a close connection between DNA methylation and cell death. (**b**) Quantitative reverse transcriptase–PCR analysis of different DNMT mRNAs showed a statistically significant upregulation of DNMT3A and DNMT3L expression in *rd1* retina. Data points in panel (**a**) represent the percentage of relative positive cells (peak value=100%)±S.D., with *n*=3–4. Bars in panel (**b**) represent mean±S.D., *n*=3, with arbitrary units (arb. un.) for mRNA expression (*wt*=1). Values were compared using the Student's *t*-test. *P<0.05, **P<0.01

To better understand the temporal dynamics of the photoreceptor DNA methylation, we analyzed 5mC immunoreactivity in *rd1* retinal sections from PN7 to PN15 and compared it with the progression of cell death as evidenced by TUNEL staining (Figure 4a). Although very low at PN7, the number of 5mC-positive cells strongly increased until PN13 (degeneration peak) to then decrease at PN15. The temporal dynamics of DNA methylation *versus rd1* TUNEL staining thus pointed to DNA methylation being a late event in photoreceptor cell death.

### DNA methylation coincides with increased DNMT expression

To test whether the observed hypermethylation was caused by increased expression of DNMTs, we applied quantitative reverse transcription-PCR (qRT-PCR) of different DNMTs in PN11 *rd1* and *wt* retinae. DNMT3A (3.5±0.9 arbitrary units (a.u.)) and DNMT3L (4.1±0.6) were found to be upregulated in the *rd1* mutants when compared with age-matched *wt* (1.0±0.2 and 1.1±0.6 a.u., respectively, *P*<0.05). No alterations in the gene expression of DNMT1 and DNMT3B were detected (Figure 4b).

To study DNA methylation also at the tissue level, we employed high-performance liquid chromatography (HPLC) coupled with mass spectrometry on global retinal samples to yield the percentage of methylated cytidines within the samples. The results suggest that from a quantitative point the DNA methylation is unchanged at the global tissue level (*wt*=3.27%±0.08; *rd1*=3.25%±0.04; *n*=5 for both genotypes). The results from this tissue-based approach thus are in contrast to the results obtained with the cellular approach above, which could indicate that the relatively low number of cells showing strong DNA methylation may not be picked up at the whole tissue level.

### DNA methylation affects different genomic regions in *rd1* and *wt*

**Figure 5:**
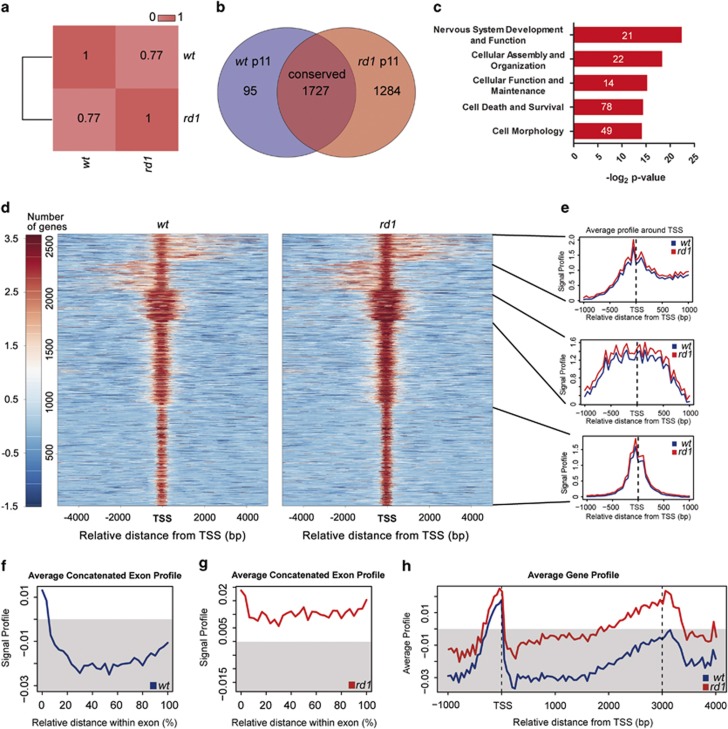
Genomic distribution of 5mC in *rd1* and *wt* mice at PN11. (**a**) Correlation plot of methylation distribution between *wt* and *rd1* retinae at PN11. Venn diagram of differentially methylated genes in *wt* (violet) and *rd1* (red), showing 1284 genes to be hypermethylated in *rd1*, whereas only 95 genes are hypomethylated. (**b**) Additionally, 1727 genes have conserved methylation patterns. (**c**) Gene ontology analysis of genes methylated in *rd1* retina. (**d**) Heatmap representations of 5mC enrichment in identified peak regions (5 kb flanking the transcription start site (TSS), which is the 5′ position of a gene sequence, where transcription starts). (**e**) Exemplified composite profiles for different clusters. Composite signal profiles of 5mC patterns in genes characterized by 5mC enrichment or lack of 5mC enrichment in concatenated exons in (**f**) the *wt* and (**g**) the *rd1* retina. The methylation profiles for *wt* and *rd1* reveal stronger methylation of exons in *rd1*, independent of the relative distance within the exon (**f** and **g**). (**h**) Overlayed average gene methylation profiles in *wt* and *rd1* retina. Note the 5mC enrichment at gene promoter regions (500 bp upstream, i.e., left of TSS) for both genotypes, and in gene body towards 3′ end of the gene in *rd1* (approximately 2000–3500 downstream, i.e., right of TSS) (**h**). Grey areas in (**f**–**h**) indicate regions without 5mC enrichment

In a qualitative approach, we assessed the genomic localization of 5mC in *wt* and *rd1* retinae by performing a DNA methylation microarray (2.1M Deluxe Promoters, NimbleGen). We used the cistrome platform (http://cistrome.org/ap/) to identify peak enrichment (MA2C tool) and performed further analysis. First, we calculated the Pearson correlation coefficients to correlate *rd1* and *wt* signal profiles that were restricted to methylated genomic regions. We detected 77% overlap of methylated regions in *rd1* and *wt*, suggesting that there are differentially methylated regions in *rd1* mice in comparison to *wt* (Figure 5a).

Mapping the peak location to genes using the peak2gene tool of the cistrome platform, we detected 1727 genes equally methylated in both groups but 95 genes that were hypomethylated in the *rd1*. However, this alteration was outbalanced by the detection of 1284 hypermethylated genes in *rd1*, which indicated increased DNA methylation in intragenic regions (Figure 5b,Supplementary Table S1). Gene ontology analysis revealed strong methylation enrichment of genes related to nervous system development and function, cellular assembly, organization, function, and maintenance as well as cell death, survival, and morphology (Figure 5c).

Moreover, *k*-means clustering revealed that methylation enrichment appeared to be stronger in specific genomic regions in the *rd1* retina (Supplementary Table S1). Example data on the distribution of average signal plots of 5mC reads on different genomic regions are visualized in Figures 5d and e. To further investigate the differential distribution on hypermethylated sites, we investigated the average exon profile in *wt* and *rd1* mice (Figures 5f and g). Remarkably, we detected stronger exonic methylation in *rd1* irrespective of its relative location within the exon. The meta-gene plot in *rd1* and *wt* (Figure 5h) revealed prominent promoter methylation in both *rd1* and *wt*. However, we also detected increased gene body methylation toward the 3′ end in *rd1* retina.

### Motif enrichment of hypermethylated *rd1* sites and gene expression correlation

**Figure 6:**
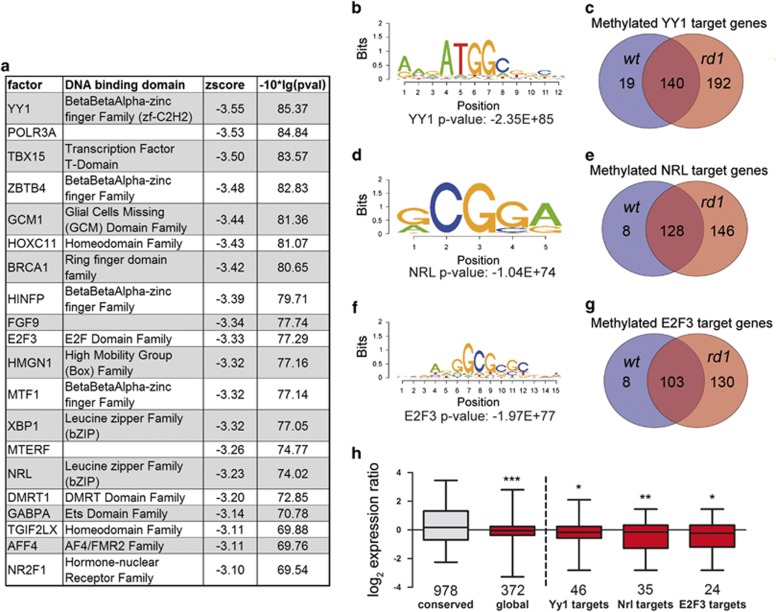
Motif enrichment of methylation patterns and gene expression correlation. (**a**) The list shows the top 20 of motifs enriched in *rd1* retina at PN11. Globally enriched motifs in methylated regions in *rd1* retina at PN11 for (**b**) YY1, (**d**) NRL, and (**f**) E2F3. Venn diagrams of methylated genes containing binding motifs for (**c**) YY1, (**e**) NRL, and (**g**) E2F3 in wt and *rd1*. (**h**) Gene expression profile of methylation unaltered (conserved) genes and hypermethylated genes in *rd1*. Bars represent mean±S.E.M., *n*=3, of log_2_ (gene) expression ratios. Pairs of groups were compared using the Student's *t*-test. *P<0.05, **P<0.01, ***P<0.005

Transcription factor binding to the DNA may be sensitive to the methylation status of its respective binding site.^21, 22^ Therefore, we used the cistrome platform (Sitepro tool) to further investigate whether 5mC was enriched in specific transcription factor-binding sites. Hypermethylation in *rd1* mice was enriched in specific binding motifs of transcription factors, including Yin Yang 1 (YY1), E2F transcription factor 3 (E2F3), and neural retina-specific leucine zipper protein (NRL) (Figures 6a–g, Supplementary Table S2). For the possible connection with RP, it is interesting to note that YY1 can interact with HDACs, is itself regulated by acetylation,^23^ and may be photoreceptor specific in the retina.^24^ E2F3, on the other hand, might contribute to rod photoreceptor cell death in the *rd1* model.^25^ Furthermore, NRL mutations are associated with autosomal-dominant RP.^26^ We thus analyzed the methylation status of potential YY1, E2F3, and NRL target genes (Supplementary Table S3). For YY1, we detected 351 target genes, of which 19 were methylated in *wt* only and 140 had a conserved methylation pattern in the transcription factor-binding sites between the two genotypes. However, the majority (192 of 351) of YY1 target genes were methylated in *rd1* only (Figure 6c). We detected a similar pattern for NRL target genes (8, 128, and 146 out of 282; *wt*, conserved, and *rd1*, respectively; Figure 6e) and E2F3 (8, 103, and 130 out of 242; *wt*, conserved, and *rd1*, respectively; Figure 6g) target genes.

We then studied the relationship between 5mC and gene expression during retinal degeneration, for which we went back to the original gene expression raw data from a previous microarray experiment^2^ and correlated this with the hypermethylated genes seen here. Global correlation analysis revealed that genes carrying methylated regions in the promoter or gene body were expressed at significantly lower levels compared with unmethylated genes (Student's *t*-test, *P*<0.005; Figure 6h). The same was true for the subgroups of YY1, E2F3, and NRL target genes (Student's *t*-test, *P*<0.05, *P*<0.01, and *P*<0.05, respectively; Figure 6h). Together, this suggested that hypermethylation of genes and their regulatory regions correlated with transcriptional repression in the *rd1* retina.

### DNMT inhibition delays *rd1* photoreceptor degeneration *in vitro*

Our study based on immunofluorescence provided strong evidence for a disease-induced increase of DNA methylation in *rd1* photoreceptors. DNMTs can be pharmacologically inhibited with cytidine-nucleotide analogues.^27, 28^ Among those, 5-aza-2'-deoxycytidine (decitabine) has been reported to reduce DNA methylation and restore gene expression patterns in human neuronal cell lines^29^ and showed promising results towards the treatment of proliferative diseases.^30, 31^ We therefore investigated whether decitabine had any effects on photoreceptor cell survival using *rd1* organotypic retinal explant cultures.

**Figure 7:**
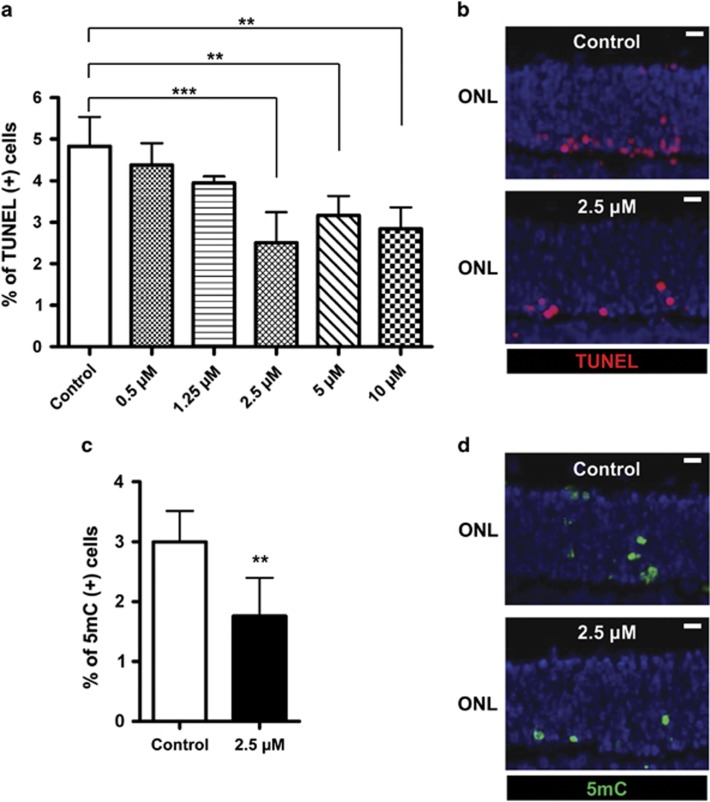
Short-term DNMT inhibition protects *rd1* photoreceptor *in vitro*. (**a** and **b**) Treatment with the DNMT inhibitor decitabine for 4 days *in vitro* significantly reduced photoreceptor cell death (expressed as the percentage of TUNEL-positive cells in the ONL). (**c** and **d**) At 2.5 *μ*M, decitabine also reduced the numbers of 5mC-positive cells. Bars represent mean±S.D., *n*=2–7, and values were compared using the Student's *t*-test. **P<0.01, ***P<0.005; ONL: the individual figures are oriented such that this layer is up and inner retinal layers are down. Scale bar=20 μm. See Material and Methods section for culturing paradigm details

Retinae from *rd1* animals received treatment with 0.5, 1.25, 2.5, 5, or 10 *μ*M decitabine for 4 days (short-term cultures, ending at postnatal (PN) day 11, Figures 7a and b; see Material and Methods section for further details on culturing paradigms). Although all concentrations tested reduced the number of TUNEL-positive cells, treatment with 2.5 *μ*M decitabine was most powerful and reduced the number of dying cells by 48% (control=4.8%±0.7; 2.5 *μ*M decitabine treated=2.5%±0.5; *P*<0.05). Quantification of the numbers of 5mC-positive cells revealed a reduction of around 41% in the treatment group compared with untreated controls (Figures 7c and d; control=3.0%±0.6; 2.5 *μ*M decitabine treated=1.8%±0.6; *P*<0.05), indicating that decitabine treatment indeed reduced DNA methylation.

We next analyzed whether DNMT inhibition promoted photoreceptor cell survival in long-term organotypic cultures treated with 2.5 *μ*M decitabine. After 12 days of treatment (ending at PN19), both groups exhibited the same number of surviving photoreceptor rows (control=3.4±0.6 rows; treated=3.4±0.3; *n*=6; *P*>0.05).

## Discussion

Our work highlights the importance of DNA methylation for neuronal cell death in the retina. Increased DNA methylation was detected in four different models for retinal degeneration representing mutations of varying kinds and with most likely various entry points into the degeneration processes. Together with earlier studies,^7^ our data points to DNA hypermethylation as a common denominator in inherited photoreceptor degeneration.

### DNA methylation and gene regulation

We found that DNA methylation was most prominent and reached ‘catastrophic' intensities during the final stages of cell death. However, we cannot exclude that hypermethylation of specific genes may be starting far earlier. For instance, we observed alterations in DNA methylation at the level of individual genes, including hypermethylation of the important transcription factors YY1, E2F3, and NRL. As we also noted a transcriptional repression of their target genes, this process should have required some time and hence the hypermethylation of the transcription factors is likely to have happened before the cell entered the final phase of cell death. Bearing in mind the nature of YY1, E2F3, and NRL target genes, this could have far-reaching effects, leading to critical dysregulation of cellular events to precipitate photoreceptor cell death.

Moreover, hypermethylation may occur in synergy with other epigenetic events, as it coincided with low acetylation, suggesting a simultaneously increased HDAC activity. This goes well with the many interconnections between DNA methylation and HDAC events,^10, 32, 33^ which may reinforce each other in preventing transcription.^34^ We therefore propose that degenerating photoreceptors trigger an epigenetic program that includes *de novo* DNA methylation to shut down transcription and protein biosynthesis to minimize energy expenditure during cell death.

Interestingly, we also noted several hypomethylated genes suggesting that demethylation of methylcytosine may also have a role during photoreceptor cell death. However, it remains elusive if demethylation is triggered by active DNA demethylation via hydroxylase activity of ten eleven translocation (Tet) enzymes, DNA repair, or oxidative damage during photoreceptor degeneration.

### DNA methylation as a novel target for retinal neuroprotection

The use of (non-decitabine) DNMT inhibitors to reduce DNA methylation has previously been proposed to be neuroprotective in motor neurons *in vivo* as well as in a neuronal cell line *in vitro*.^35^ Yet, Wang *et al.*^36^ reported neurotoxicity by decitabine in cultured dopaminergic cells. We evaluated the role of DNMTs in the degeneration process by treating organotypic *rd1* retinal explant cultures with decitabine. Although decitabine did not increase the number of surviving photoreceptors in long-term cultures, in short-term experiments it reduced DNA hypermethylation and decreased the number of dying photoreceptors. This effect of decitabine is intriguing, considering its mode of action: A cytidine analogue of decitabine is thought to be incorporated into newly synthesized DNA.^37^ This reduces the possibilities for *de novo* methylation by DNMTs in any new DNA, such as in daughter cells of proliferating cells. In addition, decitabine can promote the degradation of DNMT enzymes,^37^ to result in an overall reduction of DNA methylation.

At any rate, the protective effects of decitabine in short-term treatment confirm the causal involvement of DNA methylation in *rd1* photoreceptor cell death. As this delay in *rd1* degeneration does not manifest itself in the long term, massive DNA methylation likely occurs during the final stages of cell death.

### DNA methylation during photoreceptor cell death

Although the mechanisms of photoreceptor cell death remain elusive, in recent years the focus has shifted from apoptotic processes^38^ to non-apoptotic pathways.^39^ In particular, a number of epigenetic events have been causally associated. This includes the excessive activation of PARP1 and PARG and accumulation of PARylated proteins^3, 40, 41^ as well as over-activation of HDAC and hypoacetylation of proteins, in particular histones.^4^ Remarkably, deacetylation and excessive PARylation appeared to be occurring in sequence in the same cell, that is, deacetylation of proteins was followed by PARylation.^4^ Our current findings add DNA hypermethylation to this picture, which also appears prominently in cells that show very low protein acetylation. This corresponds to the known connections between DNA methylation and epigenetic protein modifications.^10, 32, 42^ DNA methylation and HDAC activities could, for instance, cooperate to prevent the mentioned transcription factors from acting.

The exact type of DNMT responsible for *rd1* photoreceptor DNA hypermethylation will have to be determined in future studies. DNMT1 was found to be related to survival rather than cell death, as lack of DNMT1 caused a relatively rapid photoreceptor degeneration.^43^ However, the observed upregulation of DNMT3A mRNA in the *rd1* retina suggests that this DNMT is a candidate for mediating increased methylation. This is in line with the *de novo* methyltransferase activity of DNMT3A, which could additionally complement and correct DNMT1 enzymatic activity,^12^ modifying cytosines in newly synthesized non-methylated DNA. Additionally, overexpression of DNMT3A, but not DNMT1, triggered cell death in a neuronal cell line.^35^

As mentioned above, decitabine is thought to be incorporated into newly synthesized DNA.^37^ However, the retinal photoreceptors are regarded as postmitotic neurons and are, as such, not expected to synthesize large amounts of DNA outside their development. Nevertheless, incorporation of thymidine analogs has been demonstrated in *rd1* photoreceptors, and this could reflect either ongoing DNA repair in diseased cells^44^ or a failed attempt to re-enter the cell cycle.^25^ In either case, the effect of decitabine treatment suggests that there has been an increase in DNA methylation during the *rd1* degeneration, which somehow contributes to cell death. However, it has been suggested that the execution of apoptosis is not compatible with concomitant DNA synthesis.^45^ Taken together, our findings thus point to the presence of non-apoptotic mechanisms during photoreceptor death,^39^ in which epigenetic processes have a preeminent role.

## Conclusion

In summary, we have demonstrated increased DNA methylation in photoreceptor degeneration in four different models of RP in two different species. The methylated DNA immunoprecipitation (MeDIP) and microarray data-based analyses revealed transcriptional silencing via cytosine methylation as a new player in the degeneration mechanism and provided us with fresh entry points for further investigations. Our analysis highlighted the importance of differential methylation patterns in individual *rd1* and *wt* genes. Finally, DNMT inhibition delayed retinal degeneration suggesting DNA methylation as a common denominator during photoreceptor cell death and emphasizing the potential of DNMT inhibitors for mutation-independent neuroprotection in RP.

## Materials and Methods

### Animals

Animals were kept under standard white cyclic lighting, with *ad libitum* access to food and water, and were used irrespective of gender. Four different mouse and rat mutant lines were used, together with the corresponding wild-type lines (see Table 1). P23H and S334ter rhodopsin transgenic rats were kindly provided by Dr. M M LaVail (University of California, San Francisco, CA, USA). All procedures were performed in accordance with either the Swedish (*rd1* and *rd2*, *wt* mice; permit nos. M242/07 and M220/09) and German (S334ter, P23H and CD rats, Anzeige/Mitteilung nach § 4 vom 28.04.08 and 29.04.10) animal care and ethics committees. Efforts were made to keep the number of animals used and their suffering to a minimum, and the experiments followed the ARVO statement for the use of animals in ophthalmic and visual research.

For biochemical analyses and comparisons between mutant and wild-type tissues, we used material from ages corresponding to phases where the degeneration is under way (see Table 1), but where the loss of retinal tissue is still minimal, reducing the risk for technical bias.^46, 47^ For *rd1*, this was PN 11; for *rd2* mice PN19; for *Rho* P23H rats PN15; and for *Rho* S334ter rats PN12. For the temporal analysis of 5mC detection, we used *rd1* mouse retinae from PN7, PN9, PN11, PN13, and PN15.

### Organotypic retinal explant culture

Tissue was obtained from PN5 *rd1* animals that were killed by decapitation, after which the eyes were enucleated and retinae cultured as previously described.^4, 48^ In brief, the retina and the retinal pigment epithelium (RPE) were isolated and subsequently transferred to a Millicell culture dish filter insert (Millipore AB, Solna, Sweden; PIHA03050), with the RPE layer facing the culturing membrane and incubated in R16 nutrient medium at 37 °C. The full volume of nutrient medium, 1.5 ml per dish, was replaced with fresh medium every second day (with the exception of the one-day treatment experiments below) during the culturing period.

PN5 explants were allowed to adjust to culture conditions for 2 days *in vitro*. Cultures were then treated with 5-aza-2'deoxycitydine (decitabine, no. A-3656, Sigma, Stockholm, Sweden) every second day for 4 days reaching the equivalent to PN11 (short term: PN5+2 days *in vitro*+4 days *in vitro* with treatment) or to PN19 (long term: PN5+2 days *in vitro* +12 days *in vitro* with treatment). The cultures assigned for treatment were given 0.5, 1.25, 2.5, 5, or 10 *μ*M of decitabine. Quantification of cells positive for the cell death marker TUNEL (see below) and counting of surviving photoreceptor rows represented the readout for short- and long-term cultures. All treatment experiments were done in a paired fashion, with treated and untreated samples from one animal processed together. Thus, treated/untreated samples were stained under the exact same conditions.

### Fixation, sectioning, and microscopy

Enucleated mouse and rat eyes and cultured retinae were fixed in 4% PFA in PBS at 4 °C for 2 and 1 h for mouse and rat, respectively. Eyes were cryoprotected in Sorensen's sucrose buffer and processed to 12-*μ*m cryosections.

Routine morphological observations were performed on a Zeiss Axiophot (Zeiss, Jena, Germany) microscope equipped with a Zeiss Axiocam (Zeiss) digital camera. Fluorescence excitation was provided by a HBO 100W halogen lamp. Images were taken by means of the Zeiss Axiovision 4.2 software; images elaboration and overlays were performed utilizing Adobe Photoshop CS (San Jose, CA, USA).

### Terminal dUTP nick-end labeling

TUNEL staining on fixed preparations was performed using an *in situ* kit (*In Situ* Cell Death Detection Kit; TMR Red, Roche, Mannheim, Germany). Controls with this kit and similar preparations were performed by omitting the terminal deoxynucleotidyl transferase enzyme from the labeling solution (negative control) and by pretreating the sections for 30 min with DNAse I (Roche, 3 U/ml) in 50 mM Tris-HCl, pH 7.5, 1 mg/ml BSA to induce DNA strand breaks (positive control).^3^

### Histological staining and immunofluorescence

Fixed sections were stained for general histological light microscopic analysis with hematoxylin–eosin (HE) according to the standard protocols or underwent immunostaining. For the latter, the sections were washed 3 × 5 min each in PBS containing 0.25% Triton X100 (PTX) plus 1% BSA. Blocking solution consisting of PTX with 5% normal serum from the host animal, from which the secondary antibody was obtained, was applied for 45 min. Primary antibodies were diluted in PBS with 1% BSA and 0.25% Triton X100 and applied overnight at 4 °C. Sections were then washed 3 × 5 min each in PTX and incubated with the appropriate secondary antibodies diluted in PTX for 45 min. After three more washing steps in PBS, the sections were mounted with Vectashield DAPI (Vector, Burlingame, CA, USA). Controls consisted of sections processed in parallel without primary antibody and application of the fluorescence detection system.

5mC results were obtained with the sheep anti-5mC antibody (Novus Biologicals, Cambridge, UK; category no. NB-100-744, working dilution 1 : 200) and confirmed with two other anti-5mC antibodies (Abcam, Cambridge, UK, Ab10805; 1 : 1000 and Ab51552; 1 : 50, data not shown). To exclude the possibility of unspecific antibody binding to DNA in degenerating nuclei, we also performed pretreatment of *rd1* and *wt* sections with 3 N HCl, which denatures the DNA. This yielded a weak and general nuclear staining of most retinal cells, but there was still a clear difference between the *rd1* and *wt* retinae, in that a subset of *rd1* photoreceptor nuclei attained a strong immunostaining that was not present in the *wt* situation (Supplementary Figure S1). In the 5mC staining in sections without pretreatment (see Figures 3a–d and also Figures 1c and d), there is, in principle, no positive nuclei outside of the brightly stained ones of the *rd1* ONL.

### Confocal microscopy

Samples were analyzed with a Zeiss 510 Meta confocal laser scanning microscope (Department of Biology, Lund University, Lund, Germany). The 488-nm line of a 30 mW Ar ion laser was used for AF488 excitation, and a 405-nm 15-mW solid-state laser for DAPI excitation. DAPI fluorescence was detected using a 420–480-nm bandpass filter, and AF488 with a 505–550-nm bandpass filter. A 63 × /1.5 Plan Apochromat oil immersion objective was used. The pinhole diameter was 1.0 Airy unit (giving optical sections of about 0.6 *μ*m when using the 63 × /1.5 objective). Optical sections for *z*-stacks were sampled with 50% overlap, that is, with a step size of 0.30 *μ*m. For presentation in Figures 1c and d, we used maximum intensity projections, based on 16 and 21 *Z*-sections, respectively.

### Counting of cells

The number of TUNEL- or 5mC-positive (+) cells was assessed and calculated as reported previously.^49, 50^ For each animal, at least three sections were quantified to yield an average value, and at least three different animals were analyzed for each experimental situation. Values are given as TUNEL (+) cells relative to control±S.D. Statistical significance was tested using either paired or unpaired two-tailed Student's *t*-test or ANOVA test as indicated. For all tests, a *P*-value <0.05 was considered to indicate a statistically significant difference.

### Electron microscopy

Analysis of degenerating and normal retinae was also performed at the electron microscopy (EM) level using the standard methods for transmission EM. In brief, whole eyes of mouse were enucleated and quickly punctured to improve fixative penetration. The eyes were then fixed in 2% glutaraldehyde and 2% paraformaldehyde in 0.1 M sodium cacodylate buffer overnight. The specimens were then postfixed in 1% osmium tetraoxide for 1 h, after which they were dehydrated in an ethanol series followed by acetone and embedded in Epon resin. Ultrathin sections about 50-nm thick were cut on a Leica UCT ultramicrotome (Leica Microsystems, Wetzlar, Germany), placed on single-slot grids, and stained with 2% uranylacetate for 30 min and lead citrate for 3 min. The sections were viewed in a JEOL 1230 transmission electron microscope (JEOL, Tokyo, Japan), and digital pictures with a resolution of 1024 × 1024 pixels were taken using a GATAN Multiscan camera (GATAN, Abingdon Oxon, UK).

### Methylated DNA immunoprecipitation

MeDIP was performed according to the protocol provided from Roche-NimbleGen (Madison, WI, USA). In detail, DNA was extracted from PN11 *wt* and *rd1* retinae with DNeasy kit (No. 69504, Qiagen, Hilden, Germany) according to the manufacturer's instructions. The analysis encompassed two samples of each genotype, with each sample being produced by using two retinae. DNA was incubated overnight at 37 °C with *MseI* restriction enzyme (No. R0525S, New England Biolabs, Ipswich, MA, USA), which cuts unmethylated sequences only. The buffer provided by the company was supplemented with 100 ng/*μ*l BSA, and the reaction ran overnight at 37 °C. Digested DNA samples were purified using the QIAquick PCR Purification Kit (No. 28104, Qiagen) according to the manufacturer's instructions. A volume of 1.25 *μ*l of purified DNA was then diluted in 300 *μ*l of TE buffer (10 mM Tris HCl, pH 7,5; 1 mM EDTA) and heat denatured at 95 °C for 10 min. Of this, 60 *μ*l (250 ng) were removed and stored as input control, while to the remaining DNA, 60 *μ*l of 5 × IP buffer (100 mM Na-phosphate, pH 7,0; 5 M NaCl; 10% Triton X-100; ddH_2_O until final volume) were added. Samples were immunoprecipitated using a mouse anti 5-methylcytidine antibody (Ab10805, Abcam) with a 1 : 1 ratio of antibody:DNA. The DNA:antibody mixture was incubated overnight at 4 °C on a slowly rotating platform to avoid foaming. Antibody conjugation to beads of protein A-agarose (No. 15918-014, Invitrogen, Carlsbad, CA, USA) was performed for 2 h at 4 °C by gentle rolling. After washing the beads in 1 × IP buffer, they were centrifuged at 6000 r.p.m. (=3800 × *g*) for 2 min at 4 °C, and the supernatant was discarded. Washing was repeated twice, and the beads were finally resuspended in 250-μl digestion buffer (1 M Tris HCl, pH 8,0; 0,5 M EDTA; 10% SDS; ddH_2_O until final volume). To resuspend the beads, 7 *μ*l (10 mg/ml) of proteinase K (No. 03115836011, Roche Applied Science, Penzberg, Germany) was added. Microcentrifuge tubes containing the antibody–beads complex and DNA were then sealed with Parafilm and placed in 50-ml Falcon tubes filled with damp paper towel to avoid evaporation and incubated overnight on a rotating platform at 55 °C. To purify the samples, 250 μl of phenol (No. P-4557, Sigma Aldrich) was added. Methylated DNA samples were vortexed for 30 s and centrifuged at 14 000 r.p.m. (=20800 × *g*) for 5 min at room temperature. The aqueous supernatant was saved and transferred to sterile microcentrifuge tubes. The former step was then repeated but with the addition of 250 *μ*l of chloroform:isoamyl alcohol (No. C-0549, Sigma Aldrich). To pellet DNA-purified samples, 1 *μ*l of glycogen (No. 10901393001, Roche Applied Science), 20 *μ*l of 5 M NaCl, and 500 *μ*l of ethanol were added, and precipitation of the samples occurred after incubation for 30 min at −80 °C. The pellets were centrifuged at 14 000 r.p.m. (=20800 × *g*) for 15 min at 4 °C; the supernatants were carefully removed, and samples were washed in 500 μl of cold 70% ethanol. Centrifugation was repeated, and samples were dried in a centrifugal evaporator and resuspended in 30 *μ*l of 10 mM Tris HCl (pH 8,5). We amplified 10 ng of immunoprecipitated and input DNA using the GenomePlex Complete Whole Genome Amplification (WGA) kit (No. WGA2-50RXN, Sigma Aldrich) according to the manufacturer's instructions. Finally, each sample was further purified with the Qiagen QIAquick PCR purification Kit (see step 2) according to the manufacturer's protocol. Samples were then ready for analysis by microarray (2.1M Deluxe Promoters, NimbleGen) covering >90% of the known gene promoters in the mouse DNA.

### Bioinformatics

Genome-wide binding data was analyzed with utilities in the cistrome portal.^51^ In brief, peak enrichment was analyzed by Model-based analysis of two-color arrays (MA2C) for ChIP-chip (Nimblegen) using the following parameters: bandwidth 300, max gap 250, min probes 5, threshold method *P*-value, value, 10^−6^, normalization method robust, C value 2, and mm9 assembly. Multiple wiggle file correlation was performed using methylated peak locations of *wt* and *rd1*. The Cistrome/Galaxy ‘integrative analysis – peak2gene' tool was used to retrieve all annotated genes located within 30 kb of the methylated sites and 1 kB of transcription factor-binding sites. Heatmap was generated using *k*-means cluster method and applying a *k*-means number of 5. The SitePro: Aggregation plot tool for signal profiling (version 1.0.0) was utilized to draw the average score profile around given genomic sites. Genomic location annotation and enrichment profiling was performed using CEAS (version 1.0.0.) by calculating average signal reads to generate average profile plots using the following parameters: Span 3000, Profiling resolution 50, Promoter/downstream lower-interval 1000, Promoter/downstream middle-interval 2000, Promoter/downstream upper-interval 3000, Bi-Promoter lower range 2500, Bi-Promoter upper range 5000, and Relative distance 3000. Motif analysis was performed by using the SeqPos Tool (version 1.0.0.) using a *P*-value cutoff of 0.001 and a scanning width of 600. Gene ontology analysis and pathway analysis was conducted using Database for Annotation, Visualization and Integrated Discovery. A previous microarray analysis compared the gene expression profiles of retinae from PN11 *rd1* and *wt* animals.^2^ Here, we used its BASE data to study the correlation of methylation with gene expression.

### RNA-extraction/RT-qPCR

Total RNA extraction was performed using the RNeasy-Mini Kit (Qiagen) according to the manufacturer's protocol. RNA quality was analyzed on an Agilent Bioanalyzer (Agilent Technologies, Waldbronn, Germany). RT-PCR was performed using the ThermoScript RT-PCR System (Invitrogen, Darmstadt, Germany). qPCR was performed on a LightCycler 480 System (Roche Applied Science) using KAPA SYBR FAST (Peqlab, Erlangen, Germany). Three different biological samples were analyzed in duplicates and normalized to the expression of the housekeeping gene aminolevulinic acid synthase (ALAS). Relative quantification was determined by the method described by Pfaffl.^52^ The following primers were used (5′→3′ orientation): DNMT1 fwd.: CATATCTGCAAGGACATGAG, DNMT1 rev.: CACATCATGAAAGGTCTACTG, DNMT3A fwd.: GCACGTTGGAAAGGGAGGCTGA, DNMT3A rev.: AGAAGCAGGGTCCGTGGGCT, DNMT3B fwd.: TGGCACCCTCTTCTTCATTC, DNMT3B rev.: ATATACCTTTCCAGACGCGG, DNMT3L fwd.: AGCTTGCTCCTGCTTCTGA, DNMT3L rev.:CGTGGCAGAGACTACCAGAA, ALAS fwd.: TCGCCGATGCCCATTCTTATC, and ALAS rev.: GGCCCCAACTTCCATCATCT.

Three different biological samples were analyzed in duplicates and normalized to the expression of the housekeeping gene ALAS. Relative quantification was determined.

### High-performance liquid chromatography coupled with mass spectrometry

Samples (97 *μ*l injection volume) were chromatographed by a Dionex Ultimate 3000 HPLC system (Dionex, Idstein, Germany) with a flow of 0.15 ml/min over an Uptisphere UP3HDO-150/21 column (3 *μ*m, 2.1 mm × 150 mm) from Interchim (Interchim, Mannheim, Germany). The column temperature was maintained at 30 °C. The gradient (buffer A: 0.01% formic acid in H_2_O; buffer B: 0.01% formic acid in 95% MeCN/5% H_2_O) was the following: 0→12 min; 0%→1% buffer B; 12→20 min; 1%→2% buffer B; 20→30 min; 2%→10% buffer B; 30→35 min; 10%→80% buffer B; 35→41 min; 80% buffer B; 41→51 min; 80%→0% buffer B; and 51→60 min; 0% buffer B. Sample elution was monitored at 260 nm (Dionex Ultimate 3000 Diode Array Detector). The effluent from the first 5 min (total run time of 60min) was diverted to waste by a Valco (Macherey-Nagel GmbH and Co. KG, Düren, Germany) valve to protect the mass spectrometer. The subsequent chromatographic effluent was directly injected into the ion source of a Thermo Finnigan LTQ Orbitrap XL (ThermoFinnigan, Bremen, Germany) without prior splitting. Ions were scanned using a positive polarity mode over a full-scan range of *m/z* 100–500 with a resolution of 30 000. The absolute amounts of 5mC were determined by a stable isotope dilution method and then related to the dG content by UV-detection giving the relative values in percentage. Two technical replicates per sample were performed.^53, 54, 55^

## Figures and Tables

**Table 1:** Animal strains and species used in this study; affected genes, effects, and original references

**Strain and species**	**Mutated gene**	**Effect**	**Ages used**	**Reference**
C3H wild-type mouse	—	—	PN11/PN19	^56^
C3H *rd1* mouse	*Pde6b*	cGMP accumulation, rapid rod photoreceptor degeneration, onset ≤PN10, concluded ≥PN21	PN11^a^	^13, 20^
C3H *rd2* mouse	*Prph2*	No outer segment formation, slow rod and cone degeneration, onset ≤PN16, concluded ≥PN180	PN19	^57^
CD(S.D.) wild-type rat	—	—	PN12/PN15	—
CD(S.D.) P23H rat	*Rho* (P23H tg)	Rhodopsin misfolding, rod degeneration, onset ≤PN15, concluded ≥PN90	PN15	^58^
CD(S.D.) S334ter rat	*Rho* (S334ter tg)	Rhodopsin truncation, very rapid rod degeneration, onset ≤PN10, concluded ≥PN18	PN12	^59^

a For *rd1* mice, we also used PN7, PN9, PN11, PN13, and PN15 for temporal analysis of 5mC detection

## Abbreviations

5mC: 5-methylcytosine
DMR: differentially methylated DNA regions
DNMT: DNA methyltransferase
ONL: outer nuclear layer
RP: retinitis pigmentosa
TUNEL: terminal-dUTP-nick-end-labeling
wt: wild-type
