# Supplementary figures and images for: DNA methylation and differential gene regulation in photoreceptor cell death
Source: Cell Death Dis. 2014 Dec 4;5(12):e1558–. doi: 10.1038/cddis.2014.512 (PMC4649831; doi:10.1038/cddis.2014.512)

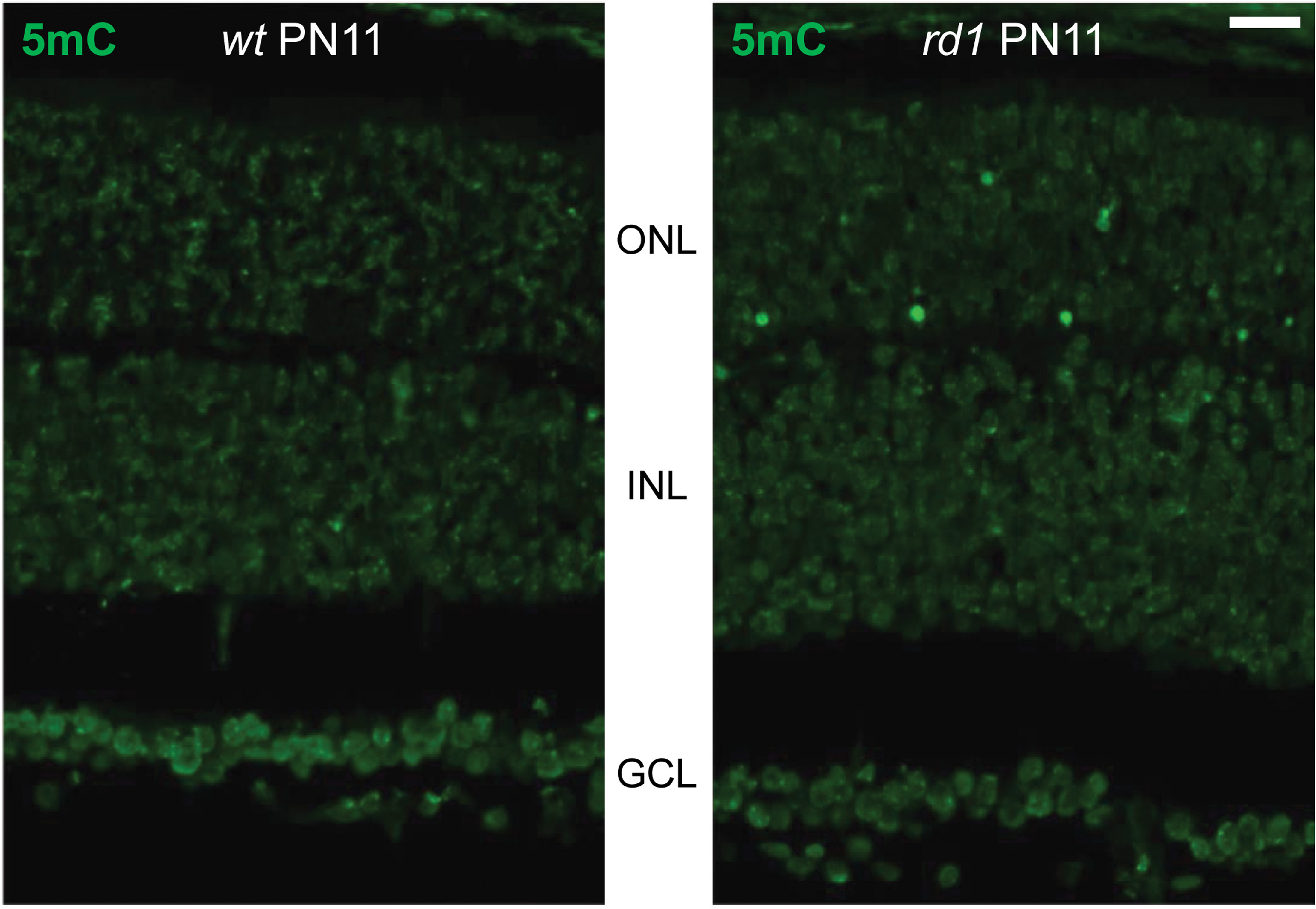

Supplement: Supplementary Figure 1 [file cddis2014512x1.tif]
